# A Case of Dysphagia, Together with Some Other Unsual Affections, Supervening an Inflammation of the Lungs, Wherein a Gum Elastic Tube Was Advantageously Employed as a Passage to the Stomach

**Published:** 1808-03

**Authors:** Edward Barlow

**Affiliations:** Member of the Royal College of Surgeons in Ireland, &c.


### *A Case of Dysphagia, together with some other unusual Affections, supervening an Inflammation of the Lungs, wherein a Gum Elastic Tube was advantageously employed as a Passage to the Stomach*; by EDWARD BARLOW, M. D. Member of the Royal College of Surgeons in Ireland, &c.

[ Extracted from the Dublin Medical and Physical Essays. ]

IT frequently happens in the practice of medicine, that a physician's embarrassment arises, not so much from the paucity of his remedies, as from the difficulty of selecting judiciously from among the multiplicity which present themselves to his choice. On the contrary, it but too often occurs, that every means of giving relief are ineffectually exhausted; and that the physician is doomed to experience that keen distress, which must ever attend the consciousness of inability to avert impending dissolution. In this latter situation, every auxiliary is valuable; and with a view to extend the knowledge of and confidence in, an important one, I trouble you with the present communication.

A lady of this city, whose age was 67, was seized, in November, 1805, with symptoms of slight pulmonic inflammation, which readily yielded to blood-letting by leeches, to blisters, and small dozes of tincture of digitalis, in conjunction with the other usual treatment. The febrile symptoms had declined, and the several others had gradually subsided. The pulse was reduced from 110 to 86, and perfectly regular; appetite was restored; and a degree of alienation of mind, which had occasionally occurred, particularly during the hot stages of the febrile exacerbations, gave place to perfect intelligence and tranquillity. Under such favourable circumstances, a speedy restoration of health was naturally expected.——On the morning, however, of Saturday, the 23d day of November, this being the seventh or eighth day of her confinement, the fourth of medical treatment, she was awakened at four o'clock by a sense of suffocation, and of inability to respire freely. By unaccountable neglect, I was not apprised of the change until twelve o'clock at noon, the hour of visiting, which I had appointed on the preceding evening, when wholly unsuspicious of any sinister event intervening. I found her much distressed by suffocation, but collected in mind, and perfectly adequate both to understand others, and to make herself intelligible, although unable to articulate culate many words in succession. This inability manifestly proceeded from the muscles of voice being either unequal to exertion, or from some other cause, disobedient to her call; and it is not unworthy of remark, that, both at this time, and throughout her subsequent illness, French words could be pronounced with apparent facility, when the correspondent English words could not be articulated. I learned from her, that she suffered no pain; had little or no cough remaining; nor any complaint, save the suffocation which I witnessed. This seemed attended by convulsive catchings of her breathing; the returns of which seemed dependent on the recurring necessities of the lungs for air, which the muscles of respiration, either from quiescence or irregular action, failed in their agency regularly to supply. The appearance was truly singular; the convulsive catchings being compounded of singultus and dyspnœa, or, in other words, being referable to irregular actions of the ordinary muscles of respiration conjointly with those of the diaphragm. These seemed to fall into irregular actions, not from a recurring disposition to such actions, as is the case in the ordinary instances of clonic spasm, but merely from the demands of the system for a renewal of the air contained within the lungs; for when by such convulsive effort fresh air was inhaled, the muscles seemed immediately to return to a state of perfect inaction, apparently the result of exhaustion and extreme debility, and to continue so until again aroused by a repetition of the same excitement; a term which I here employ solely to express that indirect influence which the necessities of the system appeared, by long established habits of association, to exert over the respiratory organs: by which influence alone these seemed, at this time, to be aroused to any exertion; extreme debility having rendered them nearly incapable of any.

As the period was critical, and the danger manifest, I called immediately for further assistance; and almost instantaneously procured the co-operation of a highly judicious physician, by whom this lady's family had been occasionally attended.

We immediately agreed on the propriety of exhibiting the camphor julep, combined with volatile fœtid spirit; and applied a large sinapism, without delay, to the fore part of the chest. By these she was so much relieved, that in the evening she took some bread and coffee with appetite, and seemed nearly free from spasm or suffocation. In order, however, to preserve the ground we had gained, and to to guard against a return of complaints, bolusses of musk, ammonia, and camphor, were ordered in addition to the former mixture, which was to be continued; due attention being also given to the essential object of nutrition.

This night she slept well, and the next morning (Sunday, 24th November) seemed refreshed. She took breakfast with appetite, but seemed not free from occasional spasms of the organs of respiration; or rather she seemed still unable to employ the respiratory muscles in that regular and continued exercise essntial to free and perfect respiration. Pills were now substituted for the boluses; these being found difficult to swallow. In the evening she grew worse, the suffocation increased, and the cough became troublesome, but without any pain, and to all appearance influenced only by the unexpectorated mucus. A sinapism was ordered, and the pills and mixture were continued. It became necessary too, at this time, to order an enema; and from the tranquilising effects she formerly experienced from the pediluvium, she was herself desirous of having this repeated, which was ordered accordingly; she also again took bread and coffee with appetite. On the following morning (Monday 25th) I visited her very early, and found her breathing still laboured, but free from pain; her pulse remained, as throughout, perfectly regular, and of good strength; she had much cough, with free expectoration, which was difficult, however, from weakness and inability to expectorate.

At eleven o'clock, A. M. deglutition became much impeded, and there was a manifestly progressive debility and tendency to spasm, extending upwards to the muscles both of voice and deglutition.

Wine was now administered, with vitriolic æther, and liquid jelly was occasionally given, in order to restore strength by every means. Notwithstanding which, a little after noon, deglutition became entirely suspended; nor could the power be restored, although the external fauces were stimulated, first with cloths wetted with vitriolic æther, and afterwards by warm oil of turpentine and tincture of cantharides. Enemeta too of asafœtida and tincture of castor were injected, with equal inefficacy.

On this evening, there being no amendment, while the strength was rapidly declining, the anxiety of the patient excessive, the throwing about of the arms almost incessant, and, in short, every prospect before us of speedy dissolution, we yet thought it right to procure the assistance of some other practitioner; and a physician of eminence was was accordingly called in, by whose advice we put sinapisms to the legs, and blisters to the soles of the feet.

The next morning (Tuesday 26th) I was deprived of the aid of that physician, who had attended with me from the onset, he being obliged to leave town. The physician, whom we last called in, however, visited with me at ten o'clock, when we had the mortification to witness a deterioration, in every respect, since the preceding evening. We found the respiration effected only by a species of singultus, and at considerable intervals; the pulse rapidly sinking; deglutition totally suspended; voice nearly lost, being capable only of uttering inarticulate monosyllables; and a countenance expressively indicative of approaching death. Still she remained perfectly sensible, was conscious of her situation, and resigned to the expected event.

Why, under such apparently hopeless circumstances, any further trials were thought expedient, may here require some explanation. In the first place, I was well assured, from the progress of the disease, and the decided concurrence of both my medical friends, that no pulmonic disease existed at this period; and that there was no ailment to contend with, save debility alone. I was satisfied that mere debility need not necessarily destroy life, so long as adequate stimulants could be employed to excite the vital powers to action; I felt that our efforts to introduce both stimulants and nutriment, had been fatally interrupted by the suspension of deglutition; and I decided in my own mind, that if by artificial deglutition a sufficient stimulus could be applied to the stomach, which organ presented the only medium through which the general system could be effectually excited, that life might even yet be preserved. With this view I had proposed, the preceding evening, the employment of a flexible tube, as a conveyance to the stomach; and had canvassed with that medical friend, whose assistance had been withdrawn me, the propriety of using such in the present case, and had received his unqualified assent, and warm approbation of the trial, as being the only means which offered any reasonable prospect of giving relief. This proposal I now repeated, and was pleased to find, that the gentleman, to whose judgment I submitted it, did not dissent; although, from the extreme hopelessnes of the case, from his being unacquainted with any similar instance, wherein such an expedient was adopted; and from some apprehension lest the attempt to introduce the tube might bring on general spasm, which might prove fatal, and be the immediate precursor precursor of death, he did not cordially coincide. I had no occasion to dwell with much force of argument on the advantages of introducing food and stimulants to the stomach; but, with respect to the objections, I pleaded that the trial was not a new experiment, as it had been repeatedly made, and with the best effects, in cases of dysphagia; that the absence of every disease, save debility, rendered the present case singularly favourable for such a trial; and, that there was little danger of exciting spasm, either local or general, by the introduction of the tube; for that, in general, it was rather by titillation, than by rougher handling, that the muscles of the fauces were thrown into irregular action; that in cases where pins were swallowed, or other extraneous matter lodged in the œsophagus, surgeons were in the constant practice of introducing *probangs* even as far as the *cardia,* without even vomiting being excited; and further, that there seemed no peculiar disposition to spasm in the present case; inasmuch as those actions, which we witnessed, were not the result of an irritable state of the muscles, so much as of a directly opposite state, a state of atony; for that they were, even with difficulty, excited to perform their necessary and accustomed actions, by the constantly recurring necessity for inspiration, and the occasional voluntary exertions to articulate; whence I inferred, that no unpleasantness of this nature would attend the operation. To all this, my medical friend yielded a reluctant assent; he agreed in its being the only expedient remaining; wished me success; and, by taking up his hat, expressively indicated his intention of not assisting in the trial; and, by this movement, effectually repressed the request for his presence and assistance, to which I was about to give utterance. He took his leave, and left me to the free exercise of my own discretion.

Embarrassing as this situation undoubtedly was, and particularly to a young practitioner, I yet resolved not to abandon the trial, but to give this last and only chance, to my old and very dear friend and relative, whose fate I sincerely lamented.

To this I was even urged by her friends, from their confidence in me, and from the simple explanation I was enabled to give of the proposed expedient. And it may serve to elucidate how far, in similar circumstances, steadiness of purpose, and conviction of the propriety of his measures, on the part of the adviser, may contribute to overcome prejudice or opposition, when I declare, that a ready ready assent was afforded to my proposition, at a time when by some strange misconception it was even understood, that the gum elastic tube was to be introduced by an artificial opening, instead of by the natural passage.

In defiance of all discouraging circumstances, I was about to proceed to the introduction, when, to my great satisfaction, I received a message from the late Dr. Purcell, whose assistance we had not been able to procure the preceding day, that he was now ready to attend, if required.

I joyfully availed myself of such valuable aid, and had soon the opportunity of detailing to him the history which I have here given. He suggested some trivial remedies; but with a candour, which I shall ever remember, he at once acknowledged, that he could propose nothing so likely to succeed, as the introduction of stimulants to the stomach by an artificial conveyance; and, after listening to my arguments and explanations, he without hesitation agreed to witness and assist me in the trial. I accordingly, under his inspection, introduced the gum elastic tube with perfect ease, and with little disturbance to our patient, while he poured in as much diluted alcohol as he deemed adviseable. The quantity, thus introduced, did not I think exceed half an ounce of a mixture containing equal parts of alcohol and water; and this he was unwilling to exceed, especially as he saw with what ease its exhibition could be repeated. After this the abdomen was fomented with warm spirits, and an enema of broth, with opium, was administered. A slight exhaustion seemed to ensue from the trifling fatigue of this inconsiderable exertion, which was soon followed, however, by a general excitement of the whole system, an increase of strength in the pulse, and more regular efforts at breathing. She lay quiet for about an hour, when, from the amendment throughout her entire appearance, and the total alteration which had taken place therein, I was induced to attempt introducing some warm wine into her mouth, with a view to her swallowing it; and, after a short interval, was delighted to find it actually swallowed, by a perceptible muscular exertion. I continued to give wine by spoonfuls, and was happy to perceive them swallowed with progressively increasing facility; at length, to my utter astonishment, she actually took the cup from my hand, and, by her own effort, raised it to her head, and drank off its contents.

This soon produced some slight aberration of mind, and a degree of incoherence, the manifest effect of a transient inebriety.

inebriety. It soon ceased, however, and on it supervened a sound and refreshing sleep, which lasted several hours, and from which she awoke apparently free from every complaint. She now with firm voice and connected expressions *asked* for wine, which was given her in conjunction with some liquid jelly, and which she took with considerable appetite. Broth enemata were repeated and retained; fomentations were again used, and she again slept soundly. On awaking the second time, the vigour both of mind and body which she evinced, astonished all her friends who witnessed it. She spoke to them severally, enquired into their health, and seemed highly gratified, both with the attentions she had received, and with the prospect of recovery, of which she had formerly desponded. Her head dress being completely unsettled, by the jactations of her body during the preceding days, she felt anxious to have it arranged; and, as it was likely to contribute to her better repose, she was indulged. During this short process, she displayed a considerable degree of liveliness and humour; and, with a *naïveté* of manner, with which she was singularly gifted, she repeated some well known lines of Pope's, on female vanity, with precision. Her head-dress and bedclothes being settled to her satisfaction, she wished for food; took some biscuit and coffee; expressed a desire to sleep, and, with that view, turned herself on her side, contracting all her limbs into the semiflex position usual with those in perfect health. Her sleep was calm and undisturbed, and continued even to seven o'clock next morning, (Wednesday the 27th,) when I was again called. Finding, however, that she had taken both wine and jelly during the night, and that a broth enema had also been given and retained, I was unwilling to disturb her, conscious that sleep was more likely to prove restorative than any means I could then employ. I returned again at ten o'clock, when I had to encounter the shock of hearing that all my hopes were fallacious, for that my friend had just expired.

Scarcely crediting the information, I entered, and found the body still warm, with a pulsation distinctly discoverable, both at the heart and wrist. Urged by the suggestions of the moment, I again introduced the tube, and poured diluted alcohol and æther into the stomach.

The stupe too, being in an adjoining chamber, I had cloths wrung from warm spirits applied to the external stomach, the abdomen, and extremities, but in vain.—At length, the spirit was fled, and not to be recalled.

It

It appeared, on enquiry, that her sleep had for some time become disturbed and uneasy, but her attendants, conceiving it injudicious to awake her, though impelled by their own feelings to do so, had suffered her to continue in this state. A few minutes before ten o'clock she awoke, apparently much agitated; breathed by short and hurried inspirations for a few moments; then breathed her last without a struggle.

It is scarcely required to offer any comments on the facts here stated; it may be right, however, to mention, that they are accurately detailed from notes regularly registered at the time of the occurrence.

The propriety of substituting an artificial mode of deglutition, when the natural had so entirely failed, can admit of little doubt,—and its success, as far as regarded the mere trial, must to all appear complete. That life was not ultimately preserved, in this case, can offer no reasonable objection; for this fatality can only lead us to infer, that, from age, and extreme delicacy of habit, the constitution was here so nicely balanced, so accurately adjusted, as though equal to the preservation of life in health, to be unable to contend with even the slightest degree of disease—and, notwithstanding the removal of disease was survived, yet, that the vital powers, exhausted by the effort, were incapable of keeping up, by any aid of stimulants, that degree of excitement necessary to the continuance of vital action. Perhaps too, we are called on to admit, that a favourable opportunity for further continuing the necessary excitement was lost, by the timidity of the attendants, who, when sleep became disturbed, and a manifest degree of agitation had commenced, would have acted more wisely by awakening her, and introducing additional wine and sustenance. With respect to the means, by which so much was accomplished, I have no hesitation in recommending its adoption in every case wherein suspended deglutition puts a stop to nutrition and medical treatment; nor do I see why its use should be confined to chronic affections, and not be extended to the latter stages of acute diseases. The expedient is simple in its nature, perfectly harmless, and applicable by any one possessed of the slightest knowledge of the parts concerned; nay, even they, who are utterly ignorant thereof, may yet employ it with safety.

Many instances of its employment, in dysphagia, are on record; and it was from recollecting one of these, detailed by the celebrated John Hunter, that I was induced to enforce force the use of it, in this case, with so much confidence. The case I here allude to, was one of dysphagia from paralytic affection of the œsophagus; and, from the means thus afforded of sustaining life, and of employing medical treatment, the disease was removed, and the patient ultimately recovered. Of the eligibility of the trial, in the present case, no doubt whatever was expressed, and of its perfect safety I had myself the fullest conviction. I trust it will appear, that I was both warranted therein by previous reasoning, and justified by the result. I further hope, that, in thus giving publicity to this detail, I do no disservice to the practice of medicine.

Conscious of the latitude which private records of diseases give for undetected embellishment, and knowing well with what distrust the unsupported assertions of individuals are oftentimes received by the public, I have deemed it incumbent on me to submit these pages to the inspection of that medical friend, who had witnessed this singular case during a principal part of its progress; and am now authorised to give, in addition to my own authority, the respectable evidence of Dr. Toole, both as to the authenticity and correctness of most of the preceding statements.

To him I am indebted for much valuable assistance during this trying attendance; and shall be, at all times, happy to express the respect which I feel both for his professional and general character. The latter part of the history, however, must rest on my own credit alone; as the corroboration of those, who witnessed it with me, could not well be asked for; and as the opportunity of adducing a still higher authority, in confirmation of this part of my statement, is unfortunately drawn from me, by the death of the late Dr. Purcell; a man, whose professional eminence was justly merited; whose urbanity of manners, and intelligence of communication, will long be remembered by all who had ever the opportunity of appreciating their value; and whose loss, every admirer of genuine worth and talents will join me in lamenting.

EDWARD BARLOW.

*Grenville-street, April* 7, 1807.

*Botanical*

